# *Leishmania* Immunity: Advancing Immunotherapy and Vaccine Development

**DOI:** 10.3390/microorganisms8081201

**Published:** 2020-08-07

**Authors:** Nnamdi M. Ikeogu, Gloria N. Akaluka, Chidalu A. Edechi, Enitan S. Salako, Chukwunonso Onyilagha, Aida F. Barazandeh, Jude E. Uzonna

**Affiliations:** 1 Department of Immunology, Max Rady College of Medicine, University of Manitoba, Winnipeg, MB R3E 0T5, Canada; akalukag@myumanitoba.ca (G.N.A.); salakoe@myumanitoba.ca (E.S.S.); chukwunonso.onyilagha@canada.ca (C.O.); feizbara@myumanitoba.ca (A.F.B.); 2 Department of Pathology, Max Rady College of Medicine, University of Manitoba, Winnipeg, MB R3E 3P5, Canada; edechia@myumanitoba.ca; 3 National Centre for Foreign Animal Disease, Canadian Food Inspection Agency, Winnipeg, MB R3E 3M4, Canada

**Keywords:** *Leishmania* infection, immunotherapy, cytokines, myeloid cells, T cells, vaccine

## Abstract

Parasitic diseases still constitute a major global health problem affecting billions of people around the world. These diseases are capable of becoming chronic and result in high morbidity and mortality. Worldwide, millions of people die each year from parasitic diseases, with the bulk of those deaths resulting from parasitic protozoan infections. Leishmaniasis, which is a disease caused by over 20 species of the protozoan parasite belonging to the genus *Leishmania*, is an important neglected disease. According to the World Health Organization (WHO), an estimated 12 million people are currently infected in about 98 countries and about 2 million new cases occur yearly, resulting in about 50,000 deaths each year. Current treatment methods for leishmaniasis are not very effective and often have significant side effects. In this review, we discussed host immunity to leishmaniasis, various treatment options currently being utilized, and the progress of both immunotherapy and vaccine development strategies used so far in leishmaniasis. We concluded with insights into what the future holds toward the fight against this debilitating parasitic disease.

## 1. Introduction

Leishmaniasis, a parasitic disease that affects humans and animals, is mainly transmitted from one host to another through the bite of an infected phlebotomine sand fly vector. Over 20 species of *Leishmania* have been reported to cause various forms of leishmaniasis, including cutaneous, mucocutaneous, and visceral forms [1,2]. Visceral leishmaniasis (VL), also known as kala-azar, is caused by *L. donovani*, and *L. infantum* [3,4]. Fever, anorexia, anemia, and weight loss accompanied by an enlarged liver and spleen are the major clinical signs and symptoms of VL, and the disease can be fatal if left untreated [2]. Mucocutaneous leishmaniasis (MCL), on the other hand, is caused by *L. panamensis*, *L. braziliensis,* and *L. guyanensis*, and is associated with a partial or complete damage of the oral, nasal, and tracheal mucosa, as well as ulcerated lesions on the skin, resulting in serious disability and lifelong scar formation [2,5]. Cutaneous leishmaniasis (CL), widely regarded as the most common form of the disease, is caused by *L. major, L. mexicana*, and *L. tropica*. This form of the disease is characterized by development of skin lesions (papules and ulcers) at the site of the sand fly bite, and the disease is usually self-resolving [2]. Like the mucocutaneous form, skin lesions caused by cutaneous leishmaniasis can leave the individual with lifelong scars and potentially lead to disability [6,7].

Members of the *Leishmania* species exist as promastigotes (extracellular, flagellated, and spindle-shaped organisms) in the sand fly vector. The promastigotes develop near the endothelial cells of the sand fly’s mid-gut [8,9]. Because sand flies are hematophagous, they inject their parasite-containing saliva into the host during a blood meal [10,11]. The injected promastigotes are engulfed by dendritic cells (DCs) or epidermal macrophages which serve as antigen presenting cells (APCs). Within the phagolysosome of these APCs, the promastigotes transform into amastigotes (which are round, immotile, and non-flagellated). Amastigotes have the capacity to survive within macrophages, proliferate by binary fission, and eventually rupture the infected cells. The parasites that are released from infected macrophages are also taken up by other macrophages to perpetuate infection in the host. Following a blood meal on an infected host, the sand fly ingests the released amastigotes, and these transform back into flagellated spindle-shaped promastigotes within the fly midgut, thus completing the parasite’s life cycle [10,11,12].

In addition to millions of new cases and thousands of deaths attributed to leishmaniasis every year, approximately 300 million people also reside in areas where the disease is endemic [2]. In addition, the disease (particularly cutaneous leishmaniasis) has been reported in tourists who visited endemic countries and Canadian and American troops returning from military operations in Afghanistan [13], thereby underscoring the importance of leishmaniasis as a public health problem. Due to the potential global spread and the health and socioeconomic impact of leishmaniasis, certain preventive and treatment measures have been established to curtail this disease. Such measures include the production of drugs for enhanced patient care, improved surveillance, and development of rapid diagnostic techniques [14]. Several treatment modalities currently employed show varying levels of efficacy and preference from region to region and between infected individuals. Pentavalent antimonial may be applied within the lesion site locally, and this has been shown to be effective in individuals suffering from cutaneous leishmaniasis [15,16,17,18]. Amphotericin-B has been employed as a reliable alternative to pentavalent antimonial in resistant VL [18,19]. Ordinarily, Amphotericin B is known to cause acute toxicity, and to avoid this issue, liposomal amphotericin B was developed. Liposomal amphotericin B is better tolerated in host cells and is as efficacious as the original amphotericin B [15,17,19]. In addition to VL, amphotericin B has also been reported to be a viable treatment option for cutaneous leishmaniasis [20]. Another drug, miltefosine, targets the promastigote and amastigote stages of the parasite and is effective against both VL and CL [21,22]. Generally, parenteral administration of antimony is the gold standard and has been very successful as a remedy for cutaneous leishmaniasis in all regions. However, the toxicity and high cost associated with the use of antimonial makes it necessary for newer treatment regimens against leishmaniasis to be developed [16,23]. As such, immunotherapy is being explored as a potential treatment strategy. In addition, there is a growing global effort towards developing a potent multispecies *Leishmania* vaccine [24], and this will largely depend on our ability to fully understand the complex interaction between *Leishmania* and its mammalian host’s immune system.

## 2. Immunity to Leishmaniasis

The successful elimination of *Leishmania* parasites from an infected host relies on the coordination of different key players of the host immune system. As the promastigotes enter into the blood stream following a sand fly bite, until the amastigotes reside within the macrophages, the tussle between the eradication of parasites and establishment of disease will be somewhat decided by the ability of *Leishmania* to modulate and/or evade host immune defenses. The components of host defense that are necessary for parasite clearance include elements of the innate and adaptive immune systems.

### 2.1. The Innate Immune Response in Leishmaniasis

#### 2.1.1. Neutrophils

In leishmaniasis, the innate immune response is mainly mediated by macrophages, dendritic cells, natural killer cells, and neutrophils. These cells interact to determine susceptibility/resistance to the infection by modulating the ensuing adaptive immune response [25,26,27]. There are divergent views on the role of neutrophils in the host immune response to leishmaniasis. At the early stages of infection, neutrophils migrate through the vasculature and are recruited to the site of infection, where they phagocytose parasites and eliminate them through a variety of mechanisms [28,29]. One such mechanism involves the use of neutrophil extracellular traps (NETs) [28,30]. The antileishmanial potency of NETs varies depending on the species of *Leishmania* involved. For instance, although NETs are effective against *L. amazonensis*, they are ineffective against *L. infantum*, *L. donovani*, and *L. mexicana* [28]. In another study, Novais et al. showed that depletion of neutrophils in vivo after *L. braziliensis* infection resulted in a remarkable increase in parasite load in infected BALB/c mice, suggesting that they contribute to resistance [14]. Nitric oxide (NO) is regarded as the major anti-*Leishmania* effector molecule in infected cells [31,32,33,34]. A study conducted by Charmoy et al. showed that neutrophils isolated from *Leishmania*-resistant C57BL/6 mice harbored significantly fewer parasites than those isolated from *Leishmania*-susceptible BALB/c mice. Furthermore, neutrophils from C57BL/6 were shown to significantly secrete more NO than those from the susceptible BALB/c mice in response to IFN-γ stimulation *in vitro*. This observation from Charmoy et al. suggests that neutrophils may contribute to anti-*Leishmania* immunity by utilizing NO-dependent mechanisms to eliminate *Leishmania* parasites [35]. In contrast, the pro-*Leishmania* activity of neutrophils was demonstrated by Peters et al., who showed that depletion of neutrophils in *L. major*-infected C57BL/6 mice resulted in decreased parasite load and diminished the progression of disease [36]. Neutrophils have also been shown to enhance the infection of macrophages by upregulating a macrophage chemokine, MIP-1β (CCL4) [37]. Neutrophils are rapidly mobilized to the site of *Leishmania* infection via *Leishmania* chemotactic factor (LCF), a selective neutrophil chemokine [38]. These neutrophils are able to phagocytose *Leishmania* and secrete Interleukin 8 (IL-8), which, in turn, increases the number of neutrophils migrating to the site of infection [39]. The infected neutrophils become apoptotic and secrete macrophage chemokines such as MIP-1β (CCL4), which leads to the chemotaxis of macrophages to the site of infection. At the site of infection, these macrophages phagocytose the infected apoptotic neutrophils. The phagocytosed *Leishmania* parasites (from the apoptotic neutrophils) are able to survive and multiply within macrophages, further establishing the disease [39]. This observation leads to neutrophils being referred to as “Trojan horses.” The participation of neutrophils in leishmaniasis has been shown to be modulated to some extent by the mode of *Leishmania* infection. Peter et al. observed massive, sustained, and localized neutrophil recruitment to the site of a sand fly bite compared to a needle injection in the dermis [40]. Taken together, these studies indicate that the role of neutrophils in host immune response to *Leishmania* infection may depend on the parasite species, mouse model used, and the mode of infection.

#### 2.1.2. Macrophages

Macrophages are also present at the site of *Leishmania* infection. Although neutrophils and macrophages are both infected by *Leishmania* parasites, macrophages serve as the major infected cells that perpetuate the infection in the host because they live longer than neutrophils [31,41,42].

As well, *Leishmania* parasites are able to promote their survival in macrophages. One mechanism by which they do so is by suppressing Interleukin 12 (IL-12) production in infected macrophages [9,43]. IL-12 is a key cytokine that induces the T-helper 1 (Th1) response which is essential for parasite elimination in infected macrophages [44,45,46]. It has been shown that the outcome of *Leishmania* infection in macrophages depends on their activation status **[42,47,48,49]**. Classical activation of macrophages is mediated by Interferon gamma (IFN-γ), an effector cytokine secreted by CD4^+^ T helper type 1 (Th1) cells, CD8^+^ T cells, and natural killer (NK) cells [50]**.** Upon IFN-γ stimulation, macrophages produce inducible nitric oxide synthase (iNOS), which converts L-arginine to nitric oxide (NO), a critical effector molecule for killing of intracellular amastigotes [50]. iNOS production is dependent on NF-κB transcription [51] and is important in facilitating the clearance of parasites. Gregory et al. reported that *Leishmania* parasites inhibit the production of NO by suppressing the production and/or activation of iNOS via *Leishmania* protease (gp63)-mediated cleavage of p65 subunit of NF- κB [51]. Also, *Leishmania* parasites inhibit NO production by increasing the expression of arginase which competitively cleaves L-arginine into ornithine [52]. Ornithine favors the proliferation of *Leishmania* parasites in macrophages [53]. Indeed, Wei et al. showed that the normally resistant (C57BL/6) mice that lost the capacity to synthesize iNOS became susceptible to *L. major* infection, although they maintained a strong IFN-γ type response [54]. An iNOS inhibitor, N(G)-monomethyl-L-arginine, suppressed IFN-γ mediated parasite clearance in both mice and human macrophages in vitro [55]. Furthermore, the administration of another iNOS inhibitor, L-N6-iminoethyl-lysine, in C57BL/6 mice, which healed their primary *L. major* infection, resulted in disease recrudescence and a 10-fold increase in parasite burden at both the draining lymph node and cutaneous site of infection. This observation suggests that continuous iNOS expression is important for primary resistance to *L. major* infection, as well as for the maintenance of infection-induced immunity in healed mice [56].

#### 2.1.3. Monocytes

Monocytes are also recruited to the site of *Leishmania* infection via chemokines (such as MIP-1β and CCL3), cytokines (such as Interleukin 8 and Tumor necrosis factor- alpha), and complement proteins, notably C5a, the breakdown product of C5 [57]. Romano et al. reported that monocytes serve as the main cell types where parasite replication occurs in vivo [58]. They further reported that early on during primary *L. major* infection, Major histocompatibility complex (MHC) II maturation of inflammatory monocytes is disrupted, unlike in secondary sites of infection. They showed that monocytes from secondary sites of infection expressed significantly higher CD86 than those from primary sites of infection. This suggests that infected monocytes at secondary sites of infection undergo extensive maturation compared to those at the primary sites of infection. This finding suggests that the in vitro infection of monocytes (as opposed to the conventional macrophage models) may be a more suitable model to study the role of *Leishmania* parasites as modulators of phagocyte maturation. This is because, in conventional macrophage models, the ability of parasites to suppress the maturation of phagocytes are assessed in already mature cells (macrophages) [58]. Furthermore, Romano et al. showed that following secondary *L. major* challenge, CCR2^+^ monocytes, which respond to CXCL10 (a chemokine produced by inflammatory cells like neutrophils in response to IFN-γ), are the major source of iNOS^+^ cells and are essential to the clearance of intracellular *L. major* parasites. The authors also suggested that neutrophils rely on CCR2^+^ monocytes for parasite clearance, as they observed that neutrophils from CCR2 depleted mice (mice which lacked monocytes) as opposed to those from CCR2 competent mice showed no evidence of leishmanicidal activity, but instead served as a safe haven for the parasites in *L. major-*infected CCR2-depleted mice.

#### 2.1.4. Dendritic Cells

Dendritic cells (DCs) are also infected by *Leishmania* and are known to be critical for antigen presentation and induction and differentiation of naïve CD4^+^ T cells into effector Th1 cells [59,60,61,62,63]. These effector Th1 cells are necessary for resistance to *Leishmania* infection [64,65,66,67]. As *Leishmania* infection progresses, DCs serve as major producers of IL-12, which is needed to prime naïve CD4^+^ T cells to differentiate into protective Th1 cells [59,60,61,62,63,68]. DCs also express costimulatory molecules such as CD40, CD80, and CD86 [69]. These costimulatory molecules have been reported to contribute to host immunity to leishmaniasis. Okwor et al. showed that antibody-mediated blockade of CD40-CD40L interaction in *L. major*-infected mice resulted in decreased production of IL-12 and IFN-γ and increased parasite burden [70]. Also, Kamanaka et al. reported that CD4- deficient C57BL/6 mice were more susceptible to *L. major* infection compared to their WT counterparts. They observed that CD40 deficiency in *L. major*-infected C57BL/6 mice tilted the Th1/Th2 balance in the favor of pro-*Leishmania* Th2 as opposed to anti-*Leishmania* Th1. This was accompanied by decreased IL-12p40 and IFN-γ response following infection [71]. In the same study, it was shown that deficiency of CD40 ligand (CD40L, normally expressed on T cell) in *L. major*-infected C57BL/6 mice resulted in a decreased Th1 immune response against *L. major* infection, leading to the development of ulcerating lesions. This increased disease severity in CD40L deficient mice was associated with inability of their Th1 cells to induce IL-12 production in macrophages. The administration of recombinant CD40L to infected CD40L deficient mice resulted in decreased lesion size and parasite burden [71]. Hathcock et al. reported that Th2 response in BALB/c mice is dependent on CD86 expression [72]. Interestingly, they also showed that the early Th1 immune response that confers protection to *L. major* in C57BL/6 relies on CD86 signaling, suggesting that the role of CD86 in leishmaniasis may be dependent on the genetic background of the mice [72].

#### 2.1.5. Natural Killer (NK) Cells

Another group of innate immune cells that play a role in immunity to leishmaniasis are NK cells [73,74,75]. Mouse infection studies showed that early (seven days post infection) depletion of NK cells decreased IFN-γ production and led to significant increase in parasite burden [76]. It was also reported that HeN mice were resistant to *L. major* infection, and this was attributed to NK cell-mediated early IFN-γ production [77]. In contrast, the enhanced susceptibility to leishmaniasis observed in BALB/c mice was associated with diminished NK cell activity [73]. Collectively, these studies suggest that NK cells contribute to host protection during *Leishmania* infection.

### 2.2. The Adaptive Immune Response in Leishmaniasis

The adaptive immune system is composed of T and B cells that mediate cell-mediated and humoral immunity, respectively. Unlike innate immunity, adaptive immunity is specific, highly specialized, and possesses immunologic memory. T cells are the major components of the host immune system that are responsible for cell-mediated immunity. Cell-mediated immunity has been shown to play a critical protective role against *Leishmania* parasites. The protective role of T cells in leishmaniasis is well established, and this was evidenced by the observation that mice that are ordinarily resistant to *Leishmania* infection became highly susceptible when their T cell response was disrupted. Furthermore, the adoptive transfer of functional T cells to these susceptible mice (with a disrupted T cell response) restored resistance to *Leishmania* infection [78].

#### 2.2.1. CD4^+^ T Cells

Various subsets of CD4^+^ T cells play different roles in leishmaniasis. IFN-γ-producing CD4^+^ Th1 cells are the major cells involved in parasite control in resistant mice. IFN-γ, which is generally regarded as the ‘signature’ Th1 cytokine, activates macrophages into an M1 phenotype, resulting in nitric oxide production and enhanced elimination of parasites resident in these macrophages [53,64,65,66,67]. The C57BL/6 mice are resistant to *L. major* because they mount a strong Th1 immune response against *Leishmania* parasites. In contrast, BALB/c mice are highly susceptible due to their relatively weaker Th1 but stronger Th2 responses [12]. Th2 cells contribute to susceptibility of BALB/c mice to leishmaniasis by producing cytokines (such as IL-4 and IL-10) that deactivate infected macrophages and suppress the iNOS pathway, which is important for eliminating intracellular *Leishmania* parasites [12]. Interestingly, the immunosuppressive effect of Th2 cells in BALB/c mice was reversed following depletion of neutrophils, and this was shown to be related to the downregulation of primary IL-4 response, thereby conferring more protection to the mice [79].

The role T-helper 17 (Th17) cells, play in immunity to *Leishmania* infection depends on the parasite species. This is because some studies have shown that Th17 cells promote susceptibility to *L. major* infection [80] but contribute to resistance to *L. infantum* [81] and *L. donovani* [82] infections. Gonzalez-Lombana et al. reported that Th17 cells enhanced host susceptibility to *L. major* infection by promoting excessive immune response [80]. On the other hand, Nascimento et al. identified IL-17A as a critical host molecule in immunity against *L. infantum*. In this study, IL-17R deficient C57BL/6 mice displayed increased susceptibility to *L. infantum* infection, as evidenced by higher parasite burden when compared to their WT counterpart mice. The increased parasite burden in IL-17R deficient C57BL/6 mice was attributed to the significant increase in IL-10-secreting regulatory T cells, coupled with a reduction in the number of CD4^+^ Th1 cells [81]. In another study, Th17 cells were also reported to be associated with resistance to *L. donovani* infection in mice due to their ability to induce chemokine secretion, which attracts neutrophils and Th1 cells to the infected sites [82].

Naturally occurring regulatory T cells (Tregs) are also found at the infected sites in cutaneous leishmaniasis, where they limit the function of effector CD4^+^ T cells through both IL-10 independent or dependent mechanisms, thus promoting long-term parasite persistence [83]. This parasite persistence is beneficial to the host because it enhances the establishment of durable immunity against the parasite [84]. This model also illustrates the importance of a host-parasite equilibrium that appears to benefit both the host and the parasite [85]. Persistence ensures the maintenance of effector memory cells that mediate infection-induced immunity in the host, as well a stable source or reservoir of parasites for transmission from one host to the other by the sand fly. In line with this, the development of lesions in mice infected with *L. major* has been associated with a high number of IL-10-secreting Tregs present at the infected site [86]. As well, the adoptive transfer of Tregs isolated from acutely infected mice to chronically infected mice was shown to lead to disease recurrence and the disruption of effector memory T cell response [87].

#### 2.2.2. CD8^+^ T Cells

Although CD4^+^ T cells are the key cells responsible for anti-*Leishmania* immunity, CD8^+^ T cells that are specific to *Leishmania* antigens have also been identified [88]. The role of CD8^+^ T cells in regulating host immune response in leishmaniasis is controversial. In one study, mice deficient in CD8^+^ T cells or class I MHC (which is necessary for CD8^+^ T cell development and activation) were able to effectively control primary *L. major* infection [89]. However, another study demonstrated that CD8^+^ T cells are crucial for resistance during low-dose parasite infection and for mounting an effective secondary immune response [88]. Uzonna et al. studied the mechanism of CD8^+^ T cell-mediated protection during low-dose infection [90]. Their study demonstrated that a transient early Th2 response was induced in C57BL/6 mice following low-dose *L. major* infection. However, this transient Th2 response was suppressed by IFN-γ produced by CD8^+^ T cells, leading to sustained Th1 response and the development of protective immunity [90]. In line with this, infection of CD8^+^ T cell-deficient C57BL/6 mice with low parasite dose resulted in persistent Th2 response and susceptibility, evidenced by increased cutaneous lesion and high parasite burden [90]. Okwor et al. also showed that although CD8^+^ T cells are selectively activated and contribute to optimal primary immunity after low-dose infection, they are dispensable during secondary immunity [91].

Whereas the role of CD8^+^ T cells in primary immunity is equivocal, their role in vaccine-induced immunity is unequivocal. Mendez et al. showed that CD8^+^ T cells are important for vaccine-induced immunity in *L. major* infection [92]. In this study, they reported that depletion of CD8^+^ T cells in vivo before vaccination with DNA expressing LACK (*Leishmania* homologue of receptors for activated C kinase), TSA (*L. major* recombinant protein homologue to eukaryotic thiol-specific-antioxidant protein), and LmSTI1 (*L. major* recombinant protein homologue to eukaryotic stress-inducible protein) compromised the ability of the vaccine to confer full protection following *L. major* challenge [92]. Although there was an observed delay in lesion development in vaccinated C57BL/6 mice treated with anti-CD8 antibody, the lesions progressed to a size comparable to unvaccinated controls [92]. In addition, CD8^+^ T cells were also shown to be important in vaccine studies with BALB/c mice. Gurunathan et al. reported that LACK DNA vaccination in conjunction with CD8^+^ T cell depletion in BALB/c mice failed to confer protection to the vaccinated BALB/c mice following *L. major* challenge [93]. When LACK DNA vaccinated mice were treated with anti-CD8, the frequency of IFN-γ producing CD4^+^ T cells were diminished both at 2 and 12 weeks post vaccination, suggesting that CD8^+^ T cells are critical for maintaining Th1 immune response in vaccinated mice [93]. Collectively, these observations indicate that CD8^+^ T cells play a critical role in vaccine-induced immunity in leishmaniasis.

#### 2.2.3. B Cells

Generally, B cells are not regarded as major contributors to protective immunity against leishmaniasis. This is because *Leishmania* are intracellular parasites and reside in the vacuole of macrophages, and as such, may not be easily accessed by antibodies [94]. To demonstrate that B cells are not protective in leishmaniasis, BALB/c mice that were sub lethally irradiated became resistant to leishmaniasis when the donor cells were CD4^+^ T cells but not when B cells were transferred [95]. Furthermore, the transfer of serum from healed mice did not confer protection [96]. However, there is some evidence to suggest that B cells may play a role in protective immune response to *Leishmania* infection. Woelbing et al. reported that IgG antibodies secreted by activated B cells in *L. major*-infected C57BL/6 mice increased antigen uptake capacity by dendritic cells via Fc gamma 3 receptor (FcγR III) [97]. This increased antigen uptake was associated with increased antigen presentation by dendritic cells, which resulted in an increased Th1 response [97]. In line with this, *L. major-*infected B cell deficient C57BL/6 mice displayed significant reduction in IFN-γ secretion and increased lesion and parasite burden, and this was reversed upon infection with IgG-opsonized parasites [97].

## 3. Advances in Vaccination against Leishmaniasis

Recovery from natural infection with most species of *Leishmania* that cause cutaneous leishmaniasis results in protection to secondary infection. This infection-induced immunity suggests that vaccination may serve as a viable option to prevent cutaneous leishmaniasis in both animals and man. Consequently, studies have been designed to determine the most effective vaccination approach against the different forms of leishmaniasis.

Whereas human vaccination studies have been disappointing, several animal vaccines appear to be effective. Vaccines, such as CaniLeish^®^ and Leishmune^®^ have shown remarkable success in the prevention of canine leishmaniasis. These vaccines, licensed for veterinary use, have been shown to confer protection in dogs and therefore prevent zoonotic transmission of leishmaniasis to humans [98]. The administration of Leishmune, a fucose-mannose ligand (FML)-based vaccine, in combination with saponin (as an adjuvant), was reported to protect 92–97% of vaccinated dogs against *L. infantum* infection [98,99]. Moreno et al. assessed the efficiency of LiESP/QA-21 vaccine on selected humoral and cellular markers of the canine immune response during the onset of immunity [100]. They showed that vaccinated dogs had significantly increased cell-mediated and humoral immune responses characterized by IFN-γ secretion and IgG2 production, respectively. These responses correlated with the induction of iNOS pathway and production of NO derivatives, which have been shown to be an important leishmanicidal mechanism [100]. A recent study with dogs in France and Spain assessed the efficacy of LetiFend^®^ (a new vaccine containing recombinant Protein Q) in protecting dogs against *L. infantum* infection. The authors showed that LetiFend^®^ can prevent the development of leishmaniasis in six-month-old dogs after natural infection with *Leishmania infantum* [101]. Recently, it was shown that the administration of recombinant ES *La*PSA-38S (rPSA) or its carboxy terminal part (*La*PSA-12S, Cter-rPSA), combined with QA-21 as adjuvant, conferred high levels of protection against *L*. *infantum* in beagles, as evidenced by a significant decrease in parasite burden and an increase in the Th1 immune response [102]

Cross protection has been reported in vaccine studies in canine leishmaniasis. Roatt et al. observed that the administration of a vaccine comprising of *L. braziliensis* antigens with saponin as an adjuvant (LBSap vaccine) conferred protection to dogs challenged with *L. infantum* mixed with salivary gland extracts. This protection was associated with significant decrease in parasite burden, and increases in IFN-γ-producing CD4^+^ and CD8^+^ T cells and total anti-*Leishmania* IgG1 and IgG2 antibody levels [103]. Also, Resende et al. showed that the administration of LbSapSal vaccine (*L*. *braziliensis* antigens, saponin as adjuvant, and *Lutzomyia longipalpis* salivary gland extract) to dogs protected the vaccinated dogs against *L*. *chagasi* challenge, as evidenced by an increase in nitric oxide production and a decreased splenic parasite load [104].

Sophisticated proteomics and cellular immunology techniques have also been employed in the search for effective *Leishmania* vaccine candidates. Using these technologies, Mou et al. identified a dominant naturally processed peptide (PEPCK_335–351_) derived from *Leishmania* glycosomal phosphoenolpyruvate carboxykinase (PEPCK) [105]. The peptide and/or its native protein elicited strong CD4^+^ T cell responses in infected mice and humans. Similar to PEPCK peptide, rPEPCK, or DNA expressing full-length PEPCK, induced strong and durable cross-species protection in both susceptible and resistant mice [105]. Recently, Lumena et al. showed that mice immunized intradermally with PEPCK were superior in generating skin-resident PEPCK-specific T cells when compared to mice immunized intramuscularly. They further observed that when challenged with *Leishmania major* parasites, intradermal immunization led to significant protection, which was not observed after intramuscular immunization [106].

Chamakh-Ayari et al. showed that the vaccination of mice with Promastigote Surface Antigen (PSA) from *L. amazonensis* (*La*PSA-38S) conferred protection to the vaccinated mice against virulent infection. Interestingly, *La*PSA-38S also induced IFN-γ secretion in human T cells *in vitro*, suggesting that its effect could be translatable to human subjects [107]. Indeed, some human vaccine trials for leishmaniasis have been conducted. In a recent study, Osman et al. described the use of ChAd63-KH, a simian adenovirus DNA-based *L. donovani* antigen vaccine, in human trial [108]. They showed that ChAd63-KH could effectively elicit strong dendritic cell activation and a wide range of CD8^+^ T cells response including IFN-γ production [108]. This study suggested that ChAd63-KH vaccine could be a potential vaccine candidate to protect humans against *L. donovani* infections [108]**.** Some vaccination strategies against leishmaniasis are summarized in Table 1.

## 4. Advances in Immunotherapy against Leishmaniasis

Immunotherapy involves the manipulation and/or activation of the immune system in order to boost or redirect the host immune response for effective therapeutic outcome. Immunotherapy for leishmaniasis is based on the principle that nonprotective anti-*Leishmania* immune response could be altered or skewed towards a protective phenotype. This could be achieved by the use of immunomodulatory agents and/or specially formulated *Leishmania* antigens. Thus, immunotherapeutic modalities may involve the use of *Leishmania* antigens, recombinant cytokines and antibodies, or molecules that modulate key immunoregulatory pathways.

### 4.1. Leishmania Antigens

A study conducted in the 1990s described the immunotherapy for patients with cutaneous leishmaniasis. Out of 62 patients that received this treatment, 42 were cured following varying rounds of administration of the *Leishmania* cocktail. Although no side effects were reported, treatment with the *Leishmania* cocktail was less effective compared to glucantime (a chemotherapeutic agent) [110]. In a relatively more recent study conducted in 2011, Jamshidi et al. compared the effect of glucantime treatment with immunotherapy consisting of killed *L. major* antigens and *Mycobacterium vaccae* as adjuvant in canine visceral leishmaniasis [111]. They found that immunotherapy alone or in combination with glucantime was more effective against canine visceral leishmaniasis when compared to chemotherapy. Although the study employed a small sample size, their findings add to the growing body of evidence supporting the potential of immunotherapy for treatment of leishmaniasis [111]. A randomized clinical trial to test the efficacy of a recombinant *Leishmania* A2 protein with saponin as adjuvant (LeishTec^®^) was conducted in the US in 2018 on dogs infected with visceral leishmaniasis. In this study, a much larger sample size of 495 dogs was utilized, and LeishTec^®^ was reported to reduce the risk of disease progression by 25% and mortality rate by 70% in treated dogs compared to controls [78]. The efficacy of vaccines produced from antigens of *L. braziliensis* in combination with a monophosphoryl lipid A (MPL) adjuvant was investigated in symptomatic dogs with visceral leishmaniasis (caused by *L. infantum*). In this study, the investigators treated 6 dogs with adjuvant alone and 10 dogs with the combination of adjuvant and *L. braziliensis* antigens [112]. The vaccine treatment led to reduction in parasite burden and disease severity. In addition, treatment with the vaccine led to enhanced anti-*Leishmania* immune response, as evidenced by elevated Th1 activity and reduced levels of *Leishmania*-promoting Th2 and IL-10 [109].

### 4.2. Cytokines and Chemokines

Cytokines and chemokines, which play key roles in immunity to *Leishmania* infection, have also been explored as potential immunotherapeutic agents. The use of recombinant IL-12 to treat *L. major*-infected BALB/c mice (at the time of infection) promoted parasite clearance and development of protective Th1 immune response [113]. Immunotherapy using recombinant CXCL-10 either in the protein form alone or expressed in *Leishmania tarentolae* (a nonpathogenic *Leishmania*) was reported to show beneficial effects against *L. major* infection in BALB/c mice [114]. The mice were treated with recombinant CXCL10 or *L. tarentolae* expressing CXCL-10 with or without adjuvant (CpG), and the effect was compared to treatment with amphotericin B. It was shown that the administration of CXCL10 expressing *L. tarentolae* along with adjuvant was effective against *L. major* although the effectiveness was less than that obtained with amphotericin B. In addition to a significant decrease in parasite burden, recombinant CXCL-10 treatment resulted in increased NO production, decreased arginase activity, and increased Th1 immune profile. Altogether, this study shows that *L. tarentolae*, a nonpathogenic specie of *Leishmania*, is a potential immunotherapeutic option for leishmaniasis and should be further explored to improve efficacy [114]. Adipokine (leptin) has also been tested as a therapeutic agent for the treatment of leishmaniasis. The administration of adipokine led to reduced parasite burden in BALB/c mice with visceral leishmaniasis, and this was associated with increased protective host immune responses, such as increased IFN-γ, IL-12, and IL-2 production. Leptin treatment was also associated with the induction of granuloma in the liver, a process that contributes to parasite killing [115].

### 4.3. Immune Checkpoint and Anti-Inflammatory Cytokine Inhibitors

Treatments which target immune suppressors such as PD-1 have been employed to control *Leishmania* infection in mouse models. In a 2019 study using BALB/c mice with nonhealing *L. amazonensis* infection, da Fonseca Martins et al. observed that anti-PD-1 and anti-PD-L1 monoclonal antibodies elicited a significant increase in IFN-γ-producing CD4^+^ and CD8^+^ T cells and resulted in increased control of infection in the BALB/c mice [116]. Quirino et al. proposed that inhibition or blockade of IL-27 production could be an interesting target for future interventions in the context of visceral leishmaniasis. This is because IL-27 negatively regulates protective IL-17 production, leading to disease exacerbation [117]. As well, inhibition of IL-10, a cytokine that is known to contribute to disease progression in leishmaniasis*,* has been reported to be beneficial in studies conducted in mouse models [118] and patients [119] with visceral leishmaniasis.

### 4.4. Inhibitors of Signalling Pathways

The use of inhibitors that target key pathways involved in immunoregulation has also shown some therapeutic potential against leishmaniasis. Khadem et al. reported that *L. major* and *L. donovani* infections can be treated through the administration of a p110δ-specific pharmacological inhibitor (CAL-101) either alone or in combination with amphotericin B [120]. They observed a significant reduction in the cutaneous lesions (*L. major*) and parasite burdens in the spleens, livers, (*L. donovani*), and footpads (*L. major*) of infected mice, coinciding with reduced regulatory T cell (Treg) numbers. Their observations present a novel option for immunotherapy and the development of drugs/vaccines against leishmaniasis [120]. Khadir et al. investigated the possibility of using mTOR inhibitors to treat *L. major* infection in mice. They found that mTOR inhibitors such as rapamycin and GSK-2126458 have immunomodulatory properties, leading to reduction in parasite load and effective control of disease progression [121]. This observation suggests that rapamycin and perhaps GSK-2126458 are potential immunotherapeutic agents for the treatment of leishmaniasis [121].

### 4.5. Modulation of Host Molecules

Host molecules may also be manipulated to influence the outcome of *Leishmania* infection. It has been reported that the deletion of liver X receptors (LXRs) gene, a host molecule that regulates the expression of genes involved in cholesterol and carbohydrate metabolism [122], resulted in enhanced leishmanicidal capacity of their macrophages, leading to a significant decrease in parasite burden compared to WT mice [123]. This study suggests that blockade of LXRs signaling may be a potential therapeutic approach in visceral leishmaniasis. Aryl hydrocarbon receptor (AhR), a transcription factor associated with inducing granuloma formation by macrophages [124], was shown to be involved in *Leishmania-*induced secretion of tumor necrosis factor (TNF) from macrophages. The administration of AhR agonist locally at the site of infection resulted in a reduced Th2 response and a decrease in disease severity [124]. This study suggests that AhR agonists may be a potential therapeutic agent against leishmaniasis. Recently, our lab showed that *L. major* infection was associated with a marked increase in the expression of long pentraxin-3 (PTX-3). The subsequent deletion of PTX-3 gene in the C57BL/6 mice resulted in enhanced resistance (reduced lesion size and parasite burden) following *L. major* infection. This was associated with significantly enhanced Th17/IL17 response, which we showed might be responsible for the enhanced resistance to *L. major* infection [125]. This study suggests that neutralization of PTX-3 in infected individuals may potentially reduce the severity of the disease. Indeed, we found significantly higher levels of PTX-3 expression in skin biopsies of patients with active skin lesion caused by *L. braziliensis* infection. Treatment with chemotherapy significantly suppressed the expression of PTX-3 in these patients [125]. In another recent study, we showed that Semaphorin-3E (Sema3E), a host molecule involved in axon guidance, was increased at the site of *L. major* infection. Targeted deletion of *Sema3E* gene resulted in increased Th1 response which might be responsible for the enhanced resistance (reduced lesion size and lower parasite burden) to *L. major* infection [126]. This study suggests that neutralization of Sema3E in infected individuals may potentially result in better disease outcome.

### 4.6. Combination Therapies

Therapeutic regimens combining immunotherapy with other agents have also been tested for the treatment of leishmaniasis. Nascimento et al. compared treatment with allopurinol alone or in combination with a subunit vaccine from *L. infantum* formulated with a second-generation lipid adjuvant (SLA) in stable emulsion (SE), referred to as (Leish-F2 + SLA-SE). Allopurinol treatment reduced the disease severity, but the effect was not long-lasting. However, a combination of Leish-F2 + SLA-SE and allopurinol was able to provide long-lasting elimination of parasites (*L. infantum*) [127]. Badaro et al. treated eight chemorefractory leishmaniasis patients with a combination of recombinant interferon gamma and conventional pentavalent antimonials. About 75% of these refractory patients responded to the combined treatment with fever as the only reported main adverse effect [15]. Ghosh et al. showed the synergistic effect of sodium antimony gluconate and dendritic cells as viable options in immunotherapy against visceral leishmaniasis. They reported that the combined administration of bone marrow-derived dendritic cells pulsed with soluble *L. donovani* antigen (SLDA) and sodium antimony gluconate resulted in complete clearance of parasites in the liver and the spleen. This was associated with Th1 immune phenotype in the treated mice [128]. Some of the immunotherapeutic agents that have been used in preclinical studies are summarized in Table 2.

## 5. Perspective and Conclusions

*Leishmania* parasites cause a range of debilitating diseases that affect millions of people and animals worldwide. This obligate intracellular organism interacts in dynamic ways with their mammalian host immune cells. *Leishmania* express thousands of antigens, have evolved to adapt to the harsh environment of the host cells, and can modulate host cell gene expression as needed. Hence, it is not surprising that they develop resistance to some currently available therapeutic agents. To complicate matters, many of the currently available drugs for treatment of leishmaniasis have serious side effects. Therefore, vaccination and immunotherapy are being considered for the treatment of leishmaniasis.

**Figure 1:**
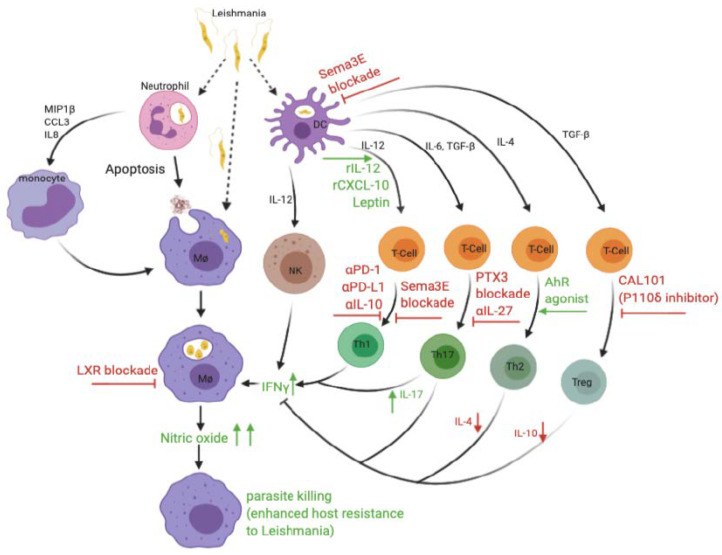
Graphical illustration of the immune response against *Leishmania* infection and some immunotherapeutic strategies to target key immune cells and molecules for treatment of Leishmaniasis.

The immune response to leishmaniasis is complex, and significant advances have been made over the years in understanding the immunopathogenesis of the disease. It is known that *Leishmania* parasites reside in macrophages and that Th1 immune response is generally considered to be the major means of parasite clearance. However, the exact role of several immune cells, such as neutrophils, Th17 cells, and CD8^+^ T cells, is still not clear. Furthermore, there is a need to better understand how *Leishmania* evades the host immune defenses and why vaccination has been relatively successful in mice and dogs but remains elusive in humans. A critical evaluation of the different vaccine studies shows that one of the possible contributing factors against successful vaccine development for humans is the use of highly susceptible BALB/c mice as a preclinical animal model for most of the vaccine design studies instead of the relatively resistant C57BL/6 mice that model human leishmaniasis. Another possible impediment to the development of human vaccine is the difficulty in finding appropriate adjuvants that can stimulate a strong cell-mediated immune response. The adjuvants that have been approved for use in humans, such as alum, MF59, ASo4, and ASo3, are poor in inducing strong and long-lasting T cell responses. A summary of key and immunotherapeutic strategies are illustrated in Figure 1.

Following inoculation into a mammalian host, *Leishmania* promastigotes infect several immune cells, including neutrophils, macrophages, and dendritic cells. The release of chemokines (e.g., MIP1β, CCL3 & IL-8) by infected neutrophils causes the recruitment of monocytes to the site of infection, and these differentiate into macrophages. In addition, infected neutrophils can also act as “Trojan horses,” thereby aiding and enhancing macrophage infection. The promastigotes develop into amastigotes in infected macrophages and perpetuate the infection. Infected dendritic cells (DCs) secrete IL-12, which activates NK cells and promotes differentiation of naïve CD4^+^ T cells into Th1 cells. Activated NK cells and Th1 cells produce IFN-γ, which stimulates production of NO by infected macrophages, leading to parasite elimination. Through currently unknown regulatory mechanisms, the production of different cytokines (such as IL-4, IL-6, and TGFβ) by infected DCs favors the differentiation of naïve CD4^+^ T cells into IL-4-producing Th2, IL-17-producing Th17, and IL-10-producing Treg cells. These cells may promote host resistance (Th17) or susceptibility (Th2, Th1,7 and Tregs) to *Leishmania*. Various immunotherapeutic strategies have been employed to boost or redirect the host immune response for effective therapeutic outcome against leishmaniasis. The blockade of LXRs leads to the death of parasite-harboring macrophages and a consequent reduction in parasite load and decreased disease severity. The administration of rCXCL-10, rIL-12, and adipokines, such as leptin, enhances IL-12 production, increases Th1 response, and increases IFN-γ and NO production, thereby promoting host resistance. The blockade of immune checkpoint inhibitors (by treatment anti-PD-1 and anti-PD-L1 mAb) or immune suppression (by anti-IL-10 mAb) increases the frequency of IFN- γ producing CD4^+^ T cells, resulting in parasite clearance. Infection with *Leishmania* (*L. major*) leads to upregulation of Sema3E and PTX3 by several immune cells. The blockade of these molecules (as demonstrated by targeted gene deletion) provides novel strategy to enhance resistance following infection. The use of agonists (e.g., AhR agonist) to block the Th2 response and the use of p110δ-specific pharmacological inhibitor (CAL-101) to inhibit the differentiation and function of Tregs are all viable immunotherapeutic approaches that have shown some promise in preclinical studies.

Significant progress has been made in identifying potential immunotherapeutic candidates against various forms of leishmaniasis. However, more work needs to be done to translate these preclinical findings from mice to humans and other animals affected by the disease. Furthermore, results from some studies reviewed herein revealed that immunotherapy is still not as effective as conventional chemotherapy. This demonstrates the need for further research to improve the efficacy of immunotherapy and further explore the possibility of combining immunotherapy with chemotherapy for better disease outcome. In conclusion, we are of the opinion that the fight against leishmaniasis will encompass both vaccination and immunotherapy as both preventive and therapeutic approaches. We also opine that both strategies need to be effectively developed further to foster a synergistic two-faced arsenal against this neglected tropical disease with a potential for global impact.

## Figures and Tables

**Table 1:** Advances in vaccination against leishmaniasis.

Vaccine Candidate	Type of Vaccine	Quality of Protection/Species	Host	Reference(s)
CaniLeish^®^	Fractionated	Good	Dogs	[98]
Leishmune^®^	Fractionated	Good	Dogs	[98,99]
LiESP/QA-21		Good	Dogs	[100]
LetiFend^®^	Recombinant protein	Good/*L. infantum*	Dogs	[101]
ES *La*PSA-38S	Recombinant protein	Good prophylactic vaccine	Dogs	[102]
LBSap vaccine (*L. brazilliensis* antigen & saponin adjuvant)	Fractionated	Good/cross-protection (*L. infantum)*	Dogs	[103]
LbSapSal vaccine (*L. brazilliensis* antigen + saponin + *lutzomiyia longpalpis* salivary gland extract)	Fractionated	Good/cross protection (*L. chagasi)*	Dogs	[104]
Leishmania glycosomal PEPCK	Recombinant protein	Good/cross protection *(L. major, L. donovani*)	Mice	[105,106]
Promastigote Surface Antigen (PSA) from *L. amazonensis*	Recombinant protein	Good/*L. amazonensis*	Human T cells	[107]
ChAd63-KH	DNA	Good/*L. donovani*	Human (Clinical trial)	[108]
*L. bralizilliensis* antigen + monophosphoryl lipid A (MPL) adjuvant	Subunit	Good/*L. infantum*	Dogs	[109]

**Table 2:** Advances in immunotherapy against leishmaniasis.

Agent	Type of Therapy	Protection	Host	Reference(s)
*Leishmania* + *M. vaccae* + Meglumine antimoniate	Immunotherapy	Effective treatment of cutaneous leishmaniasis; not as effective as glucantime	Dogs	[111]
Recombinant IL-12	Immunotherapy	Promoted parasite clearance and induced protective immunity against *L. major* challenge	Mice	[113]
Recombinant CXCL-10	Immunotherapy	Significantly decreased parasite burden, increased NO production and Th1 response	Mice	[114]
Anti-PD-1 and anti-PD-L1 monoclonal antibodies	Immunotherapy	Increased induction of protective immune cells resulting in lower parasite burden	Mice	[116]
IL-2 and IL-10 blockade by monoclonal antibody treatment	Immunotherapy	Effectively restored the host’s T cell functions leading to reduced parasite burdens	Mice	[118,119]
CAL-101 (p110δ- inhibitor)	Chemotherapy	Reduced parasite burden in cutaneous lesions, spleens, and liver	Mice	[120]
GSK-2126458 and rapamycin (m-Tor inhibitor)	Chemotherapy	Controlled disease progression and reduced parasite burden	Mice	[121]
Liver X receptors deletion	Immunotherapy	Reduced parasite burden in liver	Mice	[123]
Pentraxin-3 (PTX-3) gene deletion	Immunotherapy	Reduced cutaneous lesion and parasite burden by enhancing Th17/I17 response	Mice	[125]
Semaphorin-3E gene deletion	Immunotherapy	Reduced cutaneous lesion and parasite burden by increasing Th1 response	Mice	[126]
Allopurinol & *Leishmania* vaccine	Immunotherapy + chemotherapy	Clearance of *L. infantum* and long-lasting immunity	Mice	[127]
Soluble *L. donovani* antigen (SLDA) pulsed-BMDCs & sodium antimony gluconate	Immunotherapy + chemotherapy	Complete parasite clearance from liver and spleens	Mice	[128]
